# Sustainable choices of plant-based (‘super’) foods: examining the consumption patterns of German consumers on avocados

**DOI:** 10.3389/fnut.2023.1187626

**Published:** 2023-07-10

**Authors:** Marlene Ohlau, Sophie Christine Huning, Achim Spiller

**Affiliations:** ^1^Faculty of Agricultural Sciences, Department of Agricultural Economics and Rural Development, University of Goettingen, Göttingen, Germany; ^2^Faculty of Forest Science and Forest Ecology, University of Goettingen, Göttingen, Germany

**Keywords:** environmental awareness, plant-based diet, knowledge, food choices, dietary patterns, superfoods

## Abstract

This paper aims to better understand consumer awareness of the environmental impact of plant-based (‘super’-) foods, using avocados as an example. Since all food production impacts the environment, both meat-based and plant-based, there is scope for more sustainable food choices. Avocados have positive health properties while being considered critically for the potential negative environmental impact of their production. This study examines the avocado consumption behaviour of German consumers and the extent to which knowledge and dietary patterns are related to this. Data from 373 respondents from Germany were collected through an online consumer survey. Bivariate comparisons for avocado consumption and logistic regression analyses were performed to explore avocado consumption behaviour. The frequency of avocado consumption among respondents was moderate, averaging once per month. Avocado consumption was related to a flexitarian diet, older age and higher income, and urban dwellers. Knowledge of the environmental impacts of avocado cultivation had no influence. Respondents’ self-assessed knowledge about avocados was low. To help consumers in making conscious, sustainable choices for plant-based foods, it is necessary to provide accessible and comparable information on the environmental impact of food products. However, further life cycle assessments on avocado production are clearly needed in order to produce valid information material. A flexitarian diet with reduced consumption of animal foods is an important prerequisite for environmental sustainability. Drawing attention to environmentally friendly plant-based food choices for flexitarian consumers could further encourage them to become food innovators for a healthy planet by reducing climate impact, land use, and energy and water consumption.

## 1. Introduction

Consumers can contribute significantly to the mitigation of environmental impacts through their daily food choices (1). There is consensus among scholars that a plant-oriented diet and reduced consumption of animal-derived foods are important enablers for achieving environmental sustainability goals and staying within planetary boundaries by conserving resources (2, 3). While current food production still accounts for approximately 26% of global greenhouse gas (GHG) emissions (4), non-vegetarian diets have been found to be associated with more than twice the GHG emissions than vegetarian and vegan diets, with the difference mainly driven by meat and dairy intake (5–7). Consequently, much focus has been placed on strategies that encourage dietary shifts of consumers to plant-based foods (8, 9). However, the environmental impact of many consumer food decisions varies, even when it comes to plant-based foods. Life cycle analyses from climate and environmental science indicate that GHG emissions differ considerably between specific fruits and vegetables and their supply chains (10). For example, seasonal fruits and vegetables, such as tomatoes, carrots, and raspberries, are only considered environmentally positive when grown in their natural growing season, without the need for heating or artificial lighting in greenhouses (11–13). However, research indicates that consumers are often under- or misinformed about the various steps in food supply chains and their impact on food-related GHG emissions (14, 15). More attention has yet to be paid to public awareness towards environmentally sustainable choices of plant-based foods (16, 17).

Assessing the sustainability of plant-based food choices becomes particularly challenging when it comes to innovative products with which consumers are not yet widely familiar. In recent years, such a market has evolved with new types of plant-based foods; products referred to as ‘superfoods’ that are increasingly gaining consumer interest (18, 19). There is no legal or valid scientific definition for the term ‘superfood’, but it is used informally to refer to plant-based foods that have particularly high concentrations of vitamins, minerals, antioxidants, and secondary plant compounds (20), which have a positive impact on health and disease prevention (21–23). Therefore, it is clear that the main reason for the increasing popularity of such products is the growing consumer awareness of health and disease prevention (18, 24). What is not yet clear is the contribution of superfoods to an environmentally friendly diet. Studies addressing the environmental performance of superfoods by means of life cycle assessments^1^ (LCA) are still limited (25, 26). Nonetheless, many fruits and vegetables considered superfoods are grown and produced in countries far from where they are consumed, linking their supply chain to certain environmental impacts (19). Overall, this potentially poses a trade-off between healthy and environmentally friendly plant-based food choices, raising the question of the extent to which consumers consider environmental sustainability in addition to health considerations when choosing certain plant-based foods. Understanding consumers’ perceptions of the environmental impacts of plant-based foods, in this case superfoods, is important for developing information interventions that help consumers recognise the true impacts of their behaviours.

This paper contributes to the study of consumers’ dietary behaviours and awareness regarding the environmental impact of novel, imported plant-based (‘super’-) foods, by using avocado as an example. Global avocado consumption has increased sharply in recent years, concomitant with an intensification of production and trade. However, this has also been accompanied by a strong public debate regarding sustainability. Avocados have been the subject of controversy, as they have positive health properties (e.g. (27–30)), while also having supposedly negative effects on the environment, mainly related to high water demand, deforestation, and cultivation in monocultures (e.g. (31–34)). To date, there is no evidence that environmental concerns with avocado cultivation are reflected in consumer behaviour as the market for avocados continues to grow. Nor is there sufficient scientific research studying consumers’ avocado consumption. To the best of our knowledge, only two studies have been published on consumers’ consumption behaviour regarding avocados in the recent past. One of these studies is limited to ascertaining the effects of ripeness, maturity stage and storage damage on consumer choice in Australia (35). Going beyond that, a study by Migliore et al. (36) investigated various factors influencing avocado consumption, however, unrelated to a sustainability discourse. Therefore, this study extends previous research on avocado consumption, considering consumer awareness of and preference for environmental sustainability. In other words, a more specific understanding of the factors which are associated with avocado consumption decisions is needed.

The first objective of the present research is to describe avocado consumption among a German consumer sample. Therefore, we evaluate the consumption frequency as well as underlying motives for and against purchasing avocados. It is conceivable that health is an obvious reason for consumption, based on the beneficial macro-and micronutrients of avocado, which have already been confirmed by Migliore et al. (36). However, it would also be interesting to understand whether avocados are used as a substitute for meat, as they are an easy, unprocessed source of plant-based protein and unsaturated fatty acids (37). In this context, it could be assumed that avocado consumption is associated with certain dietary patterns, especially plant-oriented ones (i.e., flexitarians, vegetarians, and vegans). Therefore, the question arises whether consumption is connected to a particular diet. Investigating the prevalence of avocados in different diets contributes to a deeper understanding of plant-based diets and thus offers interesting insights into the consumption of potentially unsustainable foods within sustainable food groups, unveiling a potential conflict of interest.

A second objective is to determine the extent to which consumers’ knowledge of the environmental impacts of avocado production influences their consumption habits. Consumers of sustainable products generally evaluate the environmental friendliness of food products by turning to certification and labels, such as organic labels, to guide their purchase decisions (14, 38, 39). However, current food labelling systems are based on practises and indicate external process attributes or nutritional profiles, but there is a significant gap regarding the environmental impact of foods, i.e., the outcome. Several studies indicate that relevant knowledge positively affects environmental attitudes (40) and is a prerequisite to enable consumers to make environmentally friendly choices (41–43). For example, Hartmann et al. (43) have shown that consumers with higher knowledge scores are more able to compose lunch menus with a lower environmental footprint. Arguably, there is a link between consumer knowledge of the environmental impact of food and conscious, environmentally friendly food choices. Therefore, in order to assess consumer knowledge, the following questions were asked: How much knowledge do consumers have about avocado cultivation (objective knowledge) and how much do consumers think they know (subjective knowledge)?

Accordingly, the study strives to answer the following research questions:

− How is the consumption of avocados in Germany characterised, i.e., how often are avocados consumed on average, what are the motives behind consumers’ choices, and do consumption patterns adhere to certain dietary styles?
− How do objective and subjective knowledge relate to avocado consumption, i.e., are there differences in knowledge between avocado consumers and non-consumers?

## 2. Product case: avocados

### 2.1. Market situation

Global exports of avocados (*Persea Americana* Mill.) amounted to 2.5 million tonnes in 2021, on account of strong supplies from Mexico, as the leading producer accounting for nearly half of the global production, Peru and Chile (44). Avocado production within Europe originates almost entirely from Spain (44). In response to a rapidly growing global demand, avocado production has experienced the fastest growth in output in recent years and is expected to remain the fastest growing commodity of the major tropical fruits. Avocados are about to become the second-most traded major tropical fruit by 2030, after bananas (45). Its production has so far been concentrated in a small number of regions and countries, with the top 10 producing countries currently accounting for almost 80% of global output, albeit with new growing areas emerging. Nevertheless, about 74% of avocado production is expected to remain in Latin America, given the favourable growing conditions in this region (45).

Within the German market, the volume of imported avocados has increased 5-fold in 10 years, to 94,000 tonnes in 2018 (46). The fruits are mainly imported from overseas; only a small share of the market is covered by Spanish avocados (47). Since the EU signed an updated free trade agreement with Mexico in April 2020, duty-free imports of Mexican avocados into the EU have been allowed (48). This enhanced trade structure is likely to further increase the share of Mexican avocados within the EU market (49). The Netherlands are the main trade hub for avocados in Europe. Several major importers are located there, where avocados not only get distributed to many European destinations, but also are further ripened. The consumption of avocados has been fuelled by the ‘ready-to-eat’-development, as consumers generally prefer to purchase avocados when fully ripe or just before (50).

### 2.2. Sustainability

Avocados are associated with several health benefits as they provide valuable macro-and micronutrients, such as various vitamins, high concentration of potassium and magnesium, unsaturated fatty acids, and plant-based protein (29, 51). Furthermore, the avocado is highly valued for its sensory characteristics, such as taste and unique texture (52).

However, since the growing demand for avocados has led to an exponential increase in production, the debate concerning avocado and its environmental sustainability has increased in the last few years. The intensification of agriculture has produced not only economic growth but also harmful consequences for the environment and social life. Avocados are mainly grown in only a few regions of the world but consumed globally, making the impact of avocado production extremely concentrated on the planet (53).

Attention must be paid to the water consumption, as avocado is a particularly water intensive irrigated crop (54). 72% of the avocado’s edible parts consist of water (29), while avocado trees are a species that is especially sensitive to water deficits (53). The production is regionally concentrated and mainly takes place in water-scarce countries that already suffer from high water stress (53, 55). For example, the main producing countries, Chile and Mexico, are ranked 18 and 24th globally (56). As water becomes scarcer here as a result of climate change and increased agricultural use, there is growing concern about how to ensure equitable access for local communities (57–59). Water scarcity is also a growing problem for avocado production in the European region, with Spain as the main producing country increasingly suffering from water stress (ranked 28th) (56, 60).

Environmental impact of avocado production continues to arise from increased land cover changes, including deforestation and fragmentation of native forests, causing significant ecological problems (34, 61, 62). In Michoacán, a state in Mexico that accounts for 87% of avocado imports to the United States, approximately 20% of the forest has been deforested between 2001 and 2017, due to the expansion of avocado plantations (63). In addition, illegal logging and landownership conflicts occur (64, 65). The vast fragmentation and loss of forests observed to date are causing significant ecological problems (61). Cultivation in monocultures requires increased use of pesticides and fertilisers, affecting surrounding ecosystems (33, 66).

Another effect that should be considered when evaluating avocados’ sustainability is the fruit’s post-ripening during post-harvest distribution. Avocados are harvested unripe in the producing countries and then transported under refrigerated conditions by ship and container. After arriving at warehouses in Europe, avocados are ripened in chambers with controlled temperature until they reach edible ripeness (67–69). Hence, in determining the environmental impacts of avocados, it is important to consider their whole supply chains, not only the production, but also the transportation and further processing directly associated with energy consumption and GHG emissions (70, 71).

## 3. Materials and methods

### 3.1. Data collection

To address the study objectives, an online survey was conducted between November and December of 2019. To reach a wider number of participants, the snowball sampling method was used (72), taking advantage of messaging and communication platforms (e.g., Facebook, Twitter). Recruitment targeted approximately equally sized groups of avocado consumers and non-avocado consumers because of the primary objective of group comparison (Table 1).

**Table 1:** Characteristics of the total sample and of avocado consumers (AC) and non-avocado consumers (NC).

Variable	Total sample	AC	NC	χ^2^/V; t/d	*p* value
	*N* = 373	*n* = 198 (53.1%)	*n* = 175 (46.9%)		
Gender (%)					
Female	63.7%	60.0%	66.7%	χ^2^ = 2.854	
Male	36.3%	40.0%	32.8%		
Not specified	0.3%	-	0.5%		
Mean age (mean, standard deviation)	33.7 (15.4)	36.0 (16.2)	31.1 (14.1)	t = −3.162, d = −0.325	**0.002**
Level of education (%)					
Low	3.5%	3.0%	4.0%	χ^2^ = 0.051	
Middle	17.4%	16.2%	18.9%	χ^2^ = 0.300	
High	79.1%	80.8%	77.1%	χ^2^ = 0.549	
Region (%)					
Rural	22.3%	19.7%	25.1%	χ^2^ = 1.293	
Small town	22.5%	19.2%	26.3%	χ^2^ = 2.288	
Middle-sized town	15.8%	15.7%	16.0%	χ^2^ = −8.985	
Major city	39.4%	45.5%	32.6%	χ^2^ = 5.929, V = 0.126	**0.015**
Household income (%)					
Below 1,200€	36.0%	31.0%	41.7%	χ^2^ = 4.192, V = 0.106	**0.040**
1,200–3,600€	39.0%	38.1%	40.0%	χ^2^ = 0.075	
Above 3,600€	25.0%	31.0%	18.3%	χ^2^ = 7.284, V = 0.140	**0.007**
Diet (%)					
Vegan	4.3%	2.5%	6.3%	χ^2^ = 2.350	
Vegetarian	11.0%	10.6%	11.4%	χ^2^ = 0.008	
Flexitarian	35.4%	42.9%	26.9%	χ^2^ = 9.804, V = 0.162	**0.002**
Omnivore	49.3%	43.9%	55.4%	χ^2^ = 4.457, V = 0.109	**0.035**

Differences between the groups were tested using *x*^2^ chi-square test with V Cramer’s V if significant and *t* Welch’s *t*-test with *d* Cohen’s *d* if significant. Bold numbers are significantly different at *p* < 0.05.

A standardised questionnaire was developed and tested within a pre-test. In total, 379 respondents (aged ≥16 years, German speaking) completed the survey. Six respondents had to be disqualified due to inconsistent answers about their diets during data collection. Finally, the data of 373 respondents could be successfully used for further data analysis.

The questionnaire consists of four sections. First, participants provided information on socio-demographics and indicated their dietary habits as vegan, vegetarian, low-meat eater (i.e., flexitarian), or regular meat eater. Each diet was accompanied by a short explanation on how the diet is characterised. The statement was verified by asking about the frequency of meat consumption in the last month at a later stage in the survey. Second, participants’ attitudes towards sustainable consumption were inferred by them indicating their agreement with six items related to environmental characteristics (e.g., ‘I prefer to buy organically produced food’) on a Likert scale from 1 = ‘strongly disagree’ to 5 = ‘strongly agree’, based on scales developed by Steptoe et al. (73), expanded by Verain et al. (74), and Baudry et al. (75). Third, participants indicated whether they consume avocados or not. Avocado-consumers were asked about their consumption frequency of avocados, using an adapted food frequency questionnaire (FFQ) (76) as well as motives for purchasing avocados (e.g., ‘Because of the beneficial fatty acids, avocado is important for me and my health’). The fourth part related to consumer knowledge and examined the relationship between consumption behaviour regarding avocados and two aspects of consumer knowledge: objective knowledge (i.e., how much an individual actually knows about a product) and subjective knowledge (i.e., how much an individual thinks they know about a product). First, to assess participants’ objective knowledge, certain environmental impacts of avocado cultivation were stated, respondents indicated whether they had heard of them or not. Reference was made to the environmental impacts of avocado production that are most relevant and for which clear conclusions can be drawn based on the available scientific evidence. The first item refers to water consumption of avocado production and water shortages for residents in main cultivation areas, based on data from Mekonnen and Hoekstra (54), Budds (77), Hearne and Donoso (78), and DANWATCH (79) (‘The cultivation of avocados is particularly water intensive and causes water shortages for the residents in dry areas’). The second item refers to land use change based on the study from Bravo-Espinosa et al. (66) (‘Avocado production causes deforestation and fragmentation of native forests. Cultivation takes place in monocultures, which requires increased use of pesticides and fertilisers’). The third statement refers to the ripening chambers in the Netherlands, which are the transhipment point for 90% of avocados on the German market (‘Before the avocados reach the stores, they are ripened artificially in a ripening chamber’). This item is based on a report from the CBI (47). Respondents were asked to indicate whether they had heard of the main sustainable disbenefits or not by using a binary ‘yes/no’ response option. Subsequently, the respondents indicated their agreement on a scale from 1 = ‘strongly disagree’ to 5 = ‘strongly agree’ whether they have a good knowledge about avocados and nutrition in general, in order to survey the subjective knowledge.

#### 3.1.1. Ethical clearance

The study procedure was in line with the Declaration of Helsinki, and the Ethics Committee of the University of Gottingen granted ethical approval for the study.

### 3.2. Data analysis

The data analysis was performed with the statistical software package RStudio (version 2022.07.0-548). Differences between socio-demographic, dietary patterns, sustainable consumption behaviour, objective, and subjective knowledge of avocado consumers and non-avocado consumers were tested using Chi square with Cramer’s V or Welch’s *t*-test with Cohen’s *d* to estimate effect size. Avocado consumption frequency and motives for consumption were analysed using simple descriptive statistics to report percentages, means and standard deviations. Hierarchical binary logistic regression analysis was performed to identify associations with avocado consumption. The variables were entered into the model in blocks to investigate the extent to which factors predict the consumption behaviour: first, the unadjusted relationship between sociodemographic variables (gender, age, income, and region) and dietary patterns was tested. Then, the model was adjusted by adding scores on objective and subjective knowledge. Therefore, responses on subjective knowledge items were cumulated into a total score ranging from 0 (no knowledge) to 3 (complete knowledge) according to the three items. Responses towards objective knowledge were averaged into a mean score according to the two items with a scale from ‘strongly disagree’ (1) to ‘strongly agree’ (5).

## 4. Results

In total, 373 respondents were considered (Table 1). The descriptive characteristics of the dietary groups show that the proportion of women was larger in the overall sample, but decreases slightly with avocado consumption. The overall average age was 33.7, with avocado consumers being significantly older on average than non-consumers. The sample tended to be more highly educated and a larger proportion of respondents were urban dwellers. Particularly among avocado consumers, a significant majority live in metropolitan areas. Income ranged on average from 1.200 to 3.600€, with avocado consumers (AC) reporting significantly higher income than non-consumers (NC).

In terms of diet, the total sample contains 49.3% regular meat eaters (i.e., omnivores), 35.4% flexitarians, 11.0% vegetarians, and 4.3% vegans. There are significant differences between AC and NC in terms of a flexitarian diet and an omnivorous diet, with NC being more often omnivorous, while AC are more often flexitarian.

As can be seen in Table 2, just under half of the respondents who reported consuming avocados indicated a more occasional consumption, i.e., once a month or less. The main reason for purchase is taste. Followed by health, but with a scale value of 2.92 only a moderately decisive reason. As regards reasons for not consuming avocados, the main driver was also taste. Almost half of the group of non-consumers selected this as a reason not to consume avocados. Respondents also increasingly see avocado as a trend food that they would not want to follow. Environmental reasons also play a role in not consuming avocados, as well as the fact that respondents would prefer regional products.

**Table 2:** Frequency of avocado consumption and purchase motives among the group of avocado consumers (AC) as well as reasons against consuming avocados among the group of non-avocado consumers (NC).

	AC	NC
	*n* = 198 (53.1%)	*n* = 175 (46.9%)
Avocado consumption frequency (%)		
Less than once a month	48.5%	
Once a month	16.2%	
2–3 times a month	21.7%	
Once a week or more	13.6%	
Avocado consumption motives (mean, standard deviation)^1^		
Taste	3.95 (1.17)	
Health	2.92 (1.21)	
Habit	2.02 (1.12)	
Social environment	1.71 (0.97)	
Meat replacement	1.51 (0.87)	
Superfood	2.21 (1.13)	
Lifestyle	1.30 (0.62)	
Reasons for non-consumption (%)^2^		
Taste		50.9%
Trend		37.1%
Environmental impact		36.6%
Non-regional		34.3%
Price		11.4%
Availability		0.6%
Calories		0.6%

1 measured on a scale from 1 = ‘strongly disagree’ to 5 = ‘strongly agree’.

2 Share of how many respondents in the group of non-consumers selected this reason.

Table 3 shows that consumers are most likely to consider regionality when it comes to sustainability-relevant characteristics. The participants attribute medium importance to organic production and seasonality of food. Buying food with the Fairtrade label scored the lowest average approval. No significant differences were found between AC and NC in this regard.

**Table 3:** Means and standard deviation of ratings of sustainability characteristics considered by consumers when purchasing food in the total sample and between avocado consumers (AC) and non-avocado consumers (NC).

	Total sample *n* = 373	AC	NC	*t*	*p* value
		*n* = 198 (53.1%)	*n* = 175 (46.9%)		
Regionality	3.70 (0.99)	3.70 (1.02)	3.69 (0.98)	−0.102	0.919
Organic	3.29 (1.21)	3.39 (1.18)	3.18 (1.23)	−1.737	0.083
Seasonality	3.17 (0.97)	3.20 (0.91)	3.13 (1.04)	−0.756	0.450
Fairtrade certified	2.90 (1.18)	2.93 (1.13)	2.76 (1.20)	−1.593	0.154

Items were rated on a five-point scale from ‘strongly disagree’ (1) to ‘strongly agree’ (5). Differences between the groups were tested using *t* Welch’s Two Sample *t*-test.

As shown in Table 4, the most frequently reported knowledge about the consequences of avocado cultivation concerns the causation of water scarcity, followed by the aspects of land use change and the use of ripening chambers. There is no significant difference between the two groups regarding the existing objective knowledge.

**Table 4:** Proportion and differences between objective knowledge on sustainability disbenefits of the avocado production and subjective knowledge on avocado production and nutrition in general by avocado consumers (AC) and non-avocado consumers (NC).

	Total sample	AC	NC	χ^2^/*t*	*p* value
	*N* = 373	*n* = 198 (53.1%)	*n* = 175 (46.9%)		
Objective knowledge^1^					
Water scarcity	59.2%	61.1%	57.1%	χ^2^ = 0.606	0.461
Land use change	47.7%	44.9%	50.9%	χ^2^ = 1.130	0.299
Ripening chamber	36.7%	33.3%	40.6%	χ^2^ = 2.094	0.162
Subjective knowledge^2^					
Avocado production	2.33 (1.06)	2.32 (0.98)	2.34 (1.14)	*t* = 0.173	0.863
Nutrition	3.24 (0.89)	3.24 (0.88)	3.25 (0.90)	*t* = 0.090	0.928

1 Percentages, measured on a nominal scale, where 1 = ‘I know about it’ and 0 = ‘I do not know about it’; percentages indicate the response category ‘I know about it’.

2 Mean values and standard deviation, measured on a scale from 1 = ‘strongly disagree’ to 5 = ‘strongly agree’. Differences between the groups were tested using χ^2^ chi-square test and *t* Welch’s Two Sample *t*-test.

It is further shown that the respondents’ evaluation of subjective knowledge about nutrition in general tends to be in the middle range, while the subjective knowledge about avocado tends to be in the lower range, which means that self-proclaimed knowledge is rated as rather low. There is no significant difference between avocado consumers and non-consumers.

The regression analysis for avocado consumption shows that a higher age, major city residence, higher income, and a flexitarian diet significantly predict avocado consumption (Table 5). Objective and subjective knowledge, which was included subsequently into the model, did not emerge as a significant predictor and did not affect impact of the socio-demographic characteristic or diet. Accordingly, the explained variance increased marginally in the adjusted model.

**Table 5:** Results of a binary logistic regression analysis predicting consumption of avocados (*n* = 373).

	Unadjusted analysis			Adjusted analysis		
	B	SE B	OR	B	SE B	OR
Gender						
Male	0.406	0.244	1.501	0.406	0.246	1.502
Female (reference)						
Age (years, continuous)	**0.025**	**0.009**	**1.026** ^**^	**0.025**	**0.009**	**1.026** ^**^
Region						
Rural	**−0.950**	**0.317**	**0.387** ^**^	**−0.955**	**0.319**	**0.385** ^**^
Small town	**−1.176**	**0.324**	**0.309** ^***^	**−1.221**	**0.326**	**0.295** ^***^
Middle-sized town	−0.555	0.338	0.574	−0.569	0.339	0.566
Major city (reference)						
Household income						
Below 1,200€	**−0.842**	**0.376**	**0.431** ^*^	**−0.819**	**0.384**	**0.441** ^*^
1,200–3,600€	−0.513	0.307	0.599	−0.522	0.308	0.593
Above 3,600€ (reference)						
Diet						
Vegan	−0.510	0.596	0.600	−0.451	0.624	0.637
Vegetarian	0.073	0.404	1.076	0.146	0.416	1.157
Flexitarian	**0.701**	**0.266**	**2.015** ^**^	**0.754**	**0.274**	**2.126** ^**^
Omnivore (reference)						
Subjective knowledge				−0.038	0.155	0.963
Objective knowledge				−0.114	0.117	0.892
Nagelkerke R square (%)	15.5			16.0		

B, beta coefficient; SE, standard error; OR, odds ratio. Significant predictors are displayed in bold font.

^***^significant at *p* < 0.001; ^**^significant at *p* < 0.01; ^*^significant at *p* < 0.05.

## 5. Discussion

This study adds to the discussion of environmentally friendly consumption of plant-based foods, using avocado as an example, by examining underlying consumption patterns and consumer awareness of the environmental impacts of avocado production. The results showed that avocados were consumed more occasionally by the group of respondents of this study, with an average consumption of once per month.

An occasional monthly consumption can still be considered a moderate frequency of consumption. By comparison, in the United States, where half of the world’s avocado production is consumed, these fruits were shown to be consumed weekly by the majority (80). The use of these products in the habitual diet of consumers in Germany remains low for now. However, advertising the health benefits, discount offers, and the increasing plant-based lifestyle are significant drivers for a fast-rising demand and a growing market for avocados. Import numbers indicate a growing trend towards avocado consumption (81).

The taste of avocados was evaluated as the main motive for consumption. At the same time, this sensory characteristic was also the main reason against consumption. This means that this motive held more relevance than sustainability-related attributes. Health, for example, was rated only as a moderately strong motive and is thus less decisive for consumption. In published studies, the importance of sensory liking is always high (e.g. (82–85)). According to the findings of a recent nutrition report (86), the taste is the most important feature when it comes to choosing food. Yet, health is also among the key factors in food choices (e.g. (87–89)), especially in terms of fruits and vegetables (90, 91) and, also, ‘superfoods’ (18). However, the health factor is also reported to be particularly relevant when considering long-term food choices, as it is based on future-oriented intentions (85). As discovered in this study, avocados were mainly consumed occasionally, which is more in line with short-term food choices in which taste may be a superior motive. Although the taste was likewise the main reason against consumption, it is worth noting that slightly more than one-third of respondents indicated that they do not consume avocados, because they have environmental concerns and prefer regional products. Indeed, production distance seems very important for consumer perceptions of environmental and social sustainability, as confirmed by Lazzarini et al. (38). Local and seasonal products suggest authenticity and naturalness (92, 93), and it stands to reason that avocados would counter this as exotic fruits that must be imported. Regionality also showed the greatest relevance in the survey regarding the importance of sustainability-related criteria in purchasing, although non-avocado consumers do not generally purchase food with more or less sustainability orientation than avocado consumers. Nonetheless, it can be suggested that respondents are discouraged from buying avocados by some environmentally friendly attitudes.

The analysis of the drivers revealed that specific sociodemographic characteristics and dietary patterns shape avocado consumption. One of the most salient findings in this context, and relevant for sustainability assessment, is that avocado consumption is particularly associated with a flexitarian diet. On the other hand, the group of non-avocado consumers was more characterised by an omnivorous diet and, albeit not significantly, a vegan diet. A flexitarian diet is distinguished by the fact that the proportion of meat in the diet is reduced and replaced by a higher proportion of plant-based foods, thereby contributing to the sustainable development of nutrition (3, 94). Compared to meat, the water footprint of avocados is significantly less (54, 95). However, while meat production is widespread worldwide, avocados are generally grown in only a few regions but consumed globally. It follows that, unlike meat production, some environmental impacts of avocado production are extremely concentrated on certain locations (53). Making avocados not the environmentally friendliest choice within the group of plant-based products and should be consumed consciously and in a modest way. The initial assumption that avocados, as an alternative source of protein, serve as a meat substitute was contradicted by the fact that meat substitution as a specific motive for consumption was rated less applicable. Hence, it is more reasonable to assume that flexitarians consume avocados, because they reportedly enjoy trying new foods, recipes and products and place more emphasis on the variety of their diet (96). Flexitarians and plant-oriented consumers have a passionate or refined interest in foods at the forefront of trends and new diet options. Beyond that, however, studies looking at the motives, promoters and/or barriers to a reduced-meat diet also show that self-interested factors such as taste, health and nutrition rank high, sometimes higher than prosocial/ethical factors (97–99). Flexitarians have been more often reported to be less ethically motivated than vegetarians and vegans in terms of food choices (98, 100–102). Overall, this finding indicates that while flexitarians may indirectly promote an environmentally friendly diet by consuming less meat, they do not consciously make more environmentally friendly food choices regarding fruits and vegetables but rather base them on taste and health. However, flexitarians appear to be not necessarily a unified group but rather constitute different groups in which the food choices are differentiated (99, 103). Further research that specifically examines plant-based food choices in the context of a flexitarian diet would be advisable.

It was further apparent that avocado consumption was significantly related to age, urban residence, and higher income. The latter, in particular, may be indicative of the consumption of avocados, which, as ‘superfoods’, are in the higher price segment and may not appeal to all income levels. This assumption is consistent with a study by Franco Lucas et al. (24), which showed that increased consumption of superfoods is characterised by a somewhat higher income. Overall, it has often been reported that fruit and vegetable consumption is generally associated with higher income (104, 105). However, sharp price reductions for avocados can likely be expected in upcoming years, concomitant with increasing import volumes. In the long term, avocados are expected to become a standard retail product in most European countries (47). This might be a positive development, as lower food expenditure is likely to be a pivotal contributor to less healthy food choices among lower socioeconomic groups (106). However, as cultivation intensifies, it is crucial to monitor its environmental impacts.

Higher income is further assumed to correlate with a higher average age of avocado consumers. Although studies on superfoods have found that consumption and positive attitudes towards such products tend to be associated with a younger age (18, 107), these samples were older overall, whereas respondents in the present study were relatively young. An interesting finding, however, is that a large proportion of avocado consumers are located in urban regions. As described by Hawkes et al. (108), an urban food landscape can increase access to nutritious food as well as novel and unconventional food products. Contributing to this is, first, greater physical access to food. Delis and small and modern retail stores have a higher profile in the urban food landscape, providing urban populations with access to a wider variety of foods (108). Second, food choices are determined by the food environment, including the appeal and convenience of certain foods (109). Avocados are increasingly represented in restaurants and cafés, encouraging consumers to buy them. Overall, it can be assumed that avocados are more prevalent in urban dwellers’ diets due to general availability and presence and, thereby, more substantial marketing effects.

Knowledge about the environmental impact of avocado cultivation did not prove to be a significant factor influencing consumer behaviour regarding avocado consumption or abstinence. Concerning water scarcity and deforestation caused by avocado cultivation, at least half of the respondents (avocado consumers or non-consumers) stated that they had heard about it. Hence, objective knowledge about aspects of avocado cultivation was present to a certain level but without influencing whether avocados were consumed or not. This could be related to the finding that subjective knowledge was relatively low. Respondents rated their nutrition knowledge moderately, whereas avocado production knowledge was perceived to be poor. Previous work has shown that subjective knowledge affects the quality of consumers’ choices, having more influence on actual environmental behaviour than objective knowledge (110–112). It seems likely that respondents had heard of environmental impacts at some point. However, little comprehensive, substantiated information provided to consumers makes them feel insufficiently informed, resulting in deficient subjective knowledge.

In fact, there is a lack of scientifically sound information on the environmental impacts of avocado production and thus on the overall sustainability value of avocados. Missing or fragmented information leaves the consumer unable to distinguish between the specific product characteristics of avocados (e.g., country of origin, transportation mode) and, thereby, to make conscious purchasing decisions. A recent study by Jungbluth et al. (60) compared avocados from Chile, Peru, and Spain in a life cycle assessment (LCA). The LCA covers agricultural production in the country of origin, transport by ship and truck, and storage and sale. It showed that Spanish avocados cause the highest environmental impact, as Spain is one of the most water-stressed industrialised countries in the world. Although the actual water consumption for cultivation in Peru and Chile is higher, they rank comparatively lower on the water stress index. Even though the transport route by ship from South America causes more environmental impact than transport from Spain, it has less significance for the overall impacts in this analysis. This highlights how single aspects can misguide consumers’ perception of the sustainable characteristics of avocados, as, for example, consumers tend to overestimate the importance of the transport distance (15). Overall, however, this LCA is only one of very few that deal with avocados. Consequently, it is indispensable to gain further differentiated data of other producing countries to provide the necessary guidance and information to the consumer.

Some limitations of the present study should be mentioned. The sample does not represent the German population, which affects the generalisability of the results. Further research using quota sampling is needed to overcome the limitations of the external validity of the results.

To investigate the relationship between knowledge and consumption behaviour, statements were provided on environmental aspects of avocados. These refer to scientifically verified environmental impacts of avocado production, albeit on data from individual regions of cultivation. It is important to add here that production indicators vary across countries. Yet, there is no general LCA for avocado, nor is there much LCA data for individual countries. Therefore, it is important to first balance and compare further environmental and social impacts of all avocado producing countries in order to make sound recommendations.

## 6. Conclusion

A large-scale trend is currently emerging throughout society towards more sustainable diets and the consumption of plant-based foods is supposed to offer a more ethical, environmentally friendly alternative (2, 9). However, ‘superfoods’, such as avocados, are critically questioned about possible environmental impacts caused by their (expanding) production. The present study has shown that the current frequency of avocado consumption by those who participated in this study was in the moderate range. The results showed that the most relevant motive for and against consumption was taste, more important than reasons such as health benefits (pro) or environmental concerns (con). Although respondents reported having heard of some discrete environmental impacts, subjective (self-assessed) knowledge of avocado production and related environmental impacts was rated as low. Which means that consumers cannot properly evaluate and assign their (objective) knowledge. Consequently, relevant information about avocados must be provided to develop an awareness among consumers and enable them to make informed food choices. However, this is preceded by gaining more data on the sustainability assessment of avocados. So far, only a few LCA have referred to individual producing countries, which means neither a holistic assessment can be derived nor is the consumer able to distinguish between the characteristics of avocados that define their sustainability.

Strikingly, avocado consumption was significantly associated with a flexitarian diet, which is an important finding for assessing the added value of this diet for sustainability. However, it is crucial to raise awareness among those willing to eat more sustainably that conscious food choices must also be made in the context of a plant-based diet.

## Data availability statement

The raw data supporting the conclusions of this article will be made available by the authors, without undue reservation.

## Ethics statement

The studies involving human participants were reviewed and approved by Ethics Committee of the University of Gottingen. The patients/participants provided their written informed consent to participate in this study.

## Author contributions

MO, SH, and AS conceptualised the experiment. MO and SH ran the experiment. MO conducted data analysis and supervised by AS, and wrote the manuscript. MO and AS contributed to the interpretation of the results. AS revised the manuscript. All authors contributed to the article and approved the submitted version.

## Funding

The work was funded by Volkswagen Foundation, grant number ZN3382 and Ministry for Science and Culture of Lower Saxony (MWK), grant number VWZN3255. The authors are responsible for the content of this publication.

## Conflict of interest

The authors declare that the research was conducted in the absence of any commercial or financial relationships that could be construed as a potential conflict of interest.

## Publisher’s note

All claims expressed in this article are solely those of the authors and do not necessarily represent those of their affiliated organizations, or those of the publisher, the editors and the reviewers. Any product that may be evaluated in this article, or claim that may be made by its manufacturer, is not guaranteed or endorsed by the publisher.

## Footnotes

^1^Life cycle assessment addresses the potential environmental impacts and use of resources throughout a product’s life cycle from raw material acquisition through production, use, and waste management (ISO 14040:2006).
